# Maturation and Mip-1β Production of Cytomegalovirus-Specific T Cell Responses in Tanzanian Children, Adolescents and Adults: Impact by HIV and *Mycobacterium tuberculosis* Co-Infections

**DOI:** 10.1371/journal.pone.0126716

**Published:** 2015-05-14

**Authors:** Damien Portevin, Félicien Moukambi, Maxmillian Mpina, Asli Bauer, Frederick Haraka, Mkunde Chachage, Philipp Metzger, Elmar Saathoff, Petra Clowes, Nyanda E. Ntinginya, Andrea Rachow, Michael Hoelscher, Klaus Reither, Claudia A. Daubenberger, Christof Geldmacher

**Affiliations:** 1 Swiss Tropical and Public Health Institute, Basel, Switzerland; 2 University of Basel, Basel, Switzerland; 3 NIMR-Mbeya Medical Research Centre, Mbeya, Tanzania; 4 Department of Infectious Diseases and Tropical Medicine, Medical Centre of the University of Munich, Munich, Germany; 5 Ifakara Health Institute, Bagamoyo, Tanzania; 6 German Centre for Infection Research (DZIF), partner site Munich, Munich, Germany; Institute of Infection and Global Health, UNITED KINGDOM

## Abstract

It is well accepted that aging and HIV infection are associated with quantitative and functional changes of CMV-specific T cell responses. We studied here the expression of Mip-1β and the T cell maturation marker CD27 within CMVpp65-specific CD4^+^ and CD8^+^ T cells in relation to age, HIV and active Tuberculosis (TB) co-infection in a cohort of Tanzanian volunteers (≤16 years of age, n = 108 and ≥18 years, n = 79). Independent of HIV co-infection, IFNγ^+^ CMVpp65-specific CD4^+^ T cell frequencies increased with age. In adults, HIV co-infection further increased the frequencies of these cells. A high capacity for Mip-1β production together with a CD27^low^ phenotype was characteristic for these cells in children and adults. Interestingly, in addition to HIV co-infection active TB disease was linked to further down regulation of CD27 and increased capacity of Mip-1β production in CMVpp65-specific CD4^+^ T cells. These phenotypic and functional changes of CMVpp65-specific CD4 T cells observed during HIV infection and active TB could be associated with increased CMV reactivation rates.

## Introduction

Primary cytomegalovirus (CMV) infection occurs often at birth or during adolescence with prolonged viral shedding into the urine and saliva that can persist for years. In adults, primary CMV infection is typically controlled within 6 months [1]. Thereafter, CMV persists lifelong in a state of clinical latency [2,3]. The CMV-specific adaptive immune response efficiently suppresses overt viral replication and prevents viral shedding for decades. Aging is notably associated with accumulation of high frequencies of oligoclonal CMV-specific CD8^+^ T cells [4]. In late-stage AIDS patients (CD4 counts <50/μl), CMV infection can cause disseminated or localized end-organ disease [5]. CMV-specific CD4^+^ T cell responses appear to be crucial for CMV control in murine models and in humans [6],[4]. In adults, these CMV-specific CD4^+^ T cells are characterized by: (i) highly differentiated CD27^-^/CCR7^-^ effector memory phenotype; (ii) propensity to secrete the CCR5 ligand Mip-1β and (iii) high cytolytic activity against CMV infected cells and these characteristics differ from HIV- or *Vaccinia* virus-specific CD4^+^ T cells [7].

CMV-specific CD4^+^ T cells are relatively resistant to HIV infection and HIV mediated depletion [8], [9], [10], and this has been linked to their propensity to secrete Mip-1β. Mip-1β has also been connected to the expression of the immune senescence marker CD57 [11]. However, the underlying immunological mechanisms that “imprint” this particular phenotype and high capacity for Mip-1βproduction are unclear. Recent or on-going antigen exposure causes differentiation of pathogen-specific CD4^+^ T cells into CD27^-^ effector memory phenotype and can inflict functional changes [7,10,12–14]. One factor contributing to an increased capacity to produce Mip-1β could be recurrent or transient, subclinical reactivation of CMV throughout life. To address this hypothesis, we studied cell frequencies, Mip-1β production and CD27 expression of CMV-specific CD4^+^ and CD8^+^ T cells in children, adolescence and adults in relation to age, HIV and active tuberculosis infection in individuals from Tanzania, where CMV infection prevalence reaches up to 100% in adults [15].

## Results and Discussion

**Fig 1:**
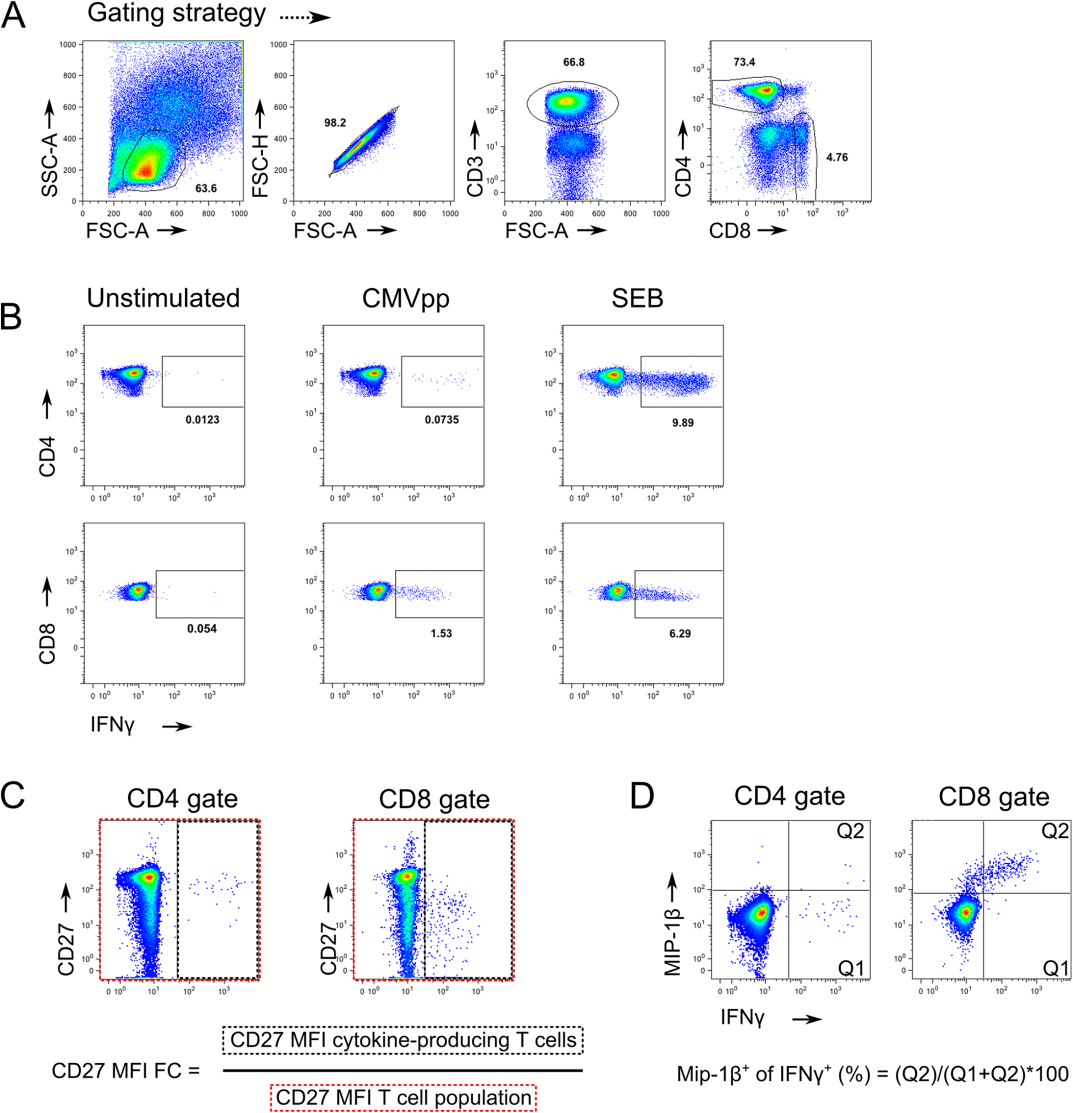
Gating strategies and CMV responses evaluation. A) Morphological and CD marker expression gating strategy of CD4 and CD8 T cell. B) Representative CD4 and CD8 T cell IFNγ response from a significant responder. C) Gating strategy for the CD27 MFI Fold Change calculation of IFNγ producing cells compared to all CD4 or CD8 T cells. D) Gating strategy for the determination of IFNγ producing cells that were also positive for Mip1β production.

Detection of CMV-specific T cell responses using polychromatic flow cytometry after in vitro stimulation is a reliable marker for determining the CMV infection status in children [16]. CMVpp65-specific CD4^+^ and CD8^+^ T cell responses were characterized in a Tanzanian cohort (n = 187) encompassing children (<10 years of age, n = 79), adolescents (10–16 years of age, n = 29) and adults (>18 years of age, n = 79). This Tanzanian cohort is described in greater detail [17]. The gating strategy and representative flow cytometry plots are shown in Fig 1. Study subjects were stratified according to age and their HIV and TB co-infection status. In the absence of a reliable test for definite childhood TB classification, HIV^-^ paediatric TB suspects included culture-confirmed TB (n = 14) and uncertain TB classification (n = 16). HIV^+^ paediatric TB suspects (n = 31) included culture confirmed TB (n = 4), and 13 subjects with uncertain TB classification (n = 13). In 46 HIV^-^ and 13 HIV^+^ children an alternative diagnosis could be established after 5 months of clinical follow-up excluding active TB [17].

**Fig 2:**
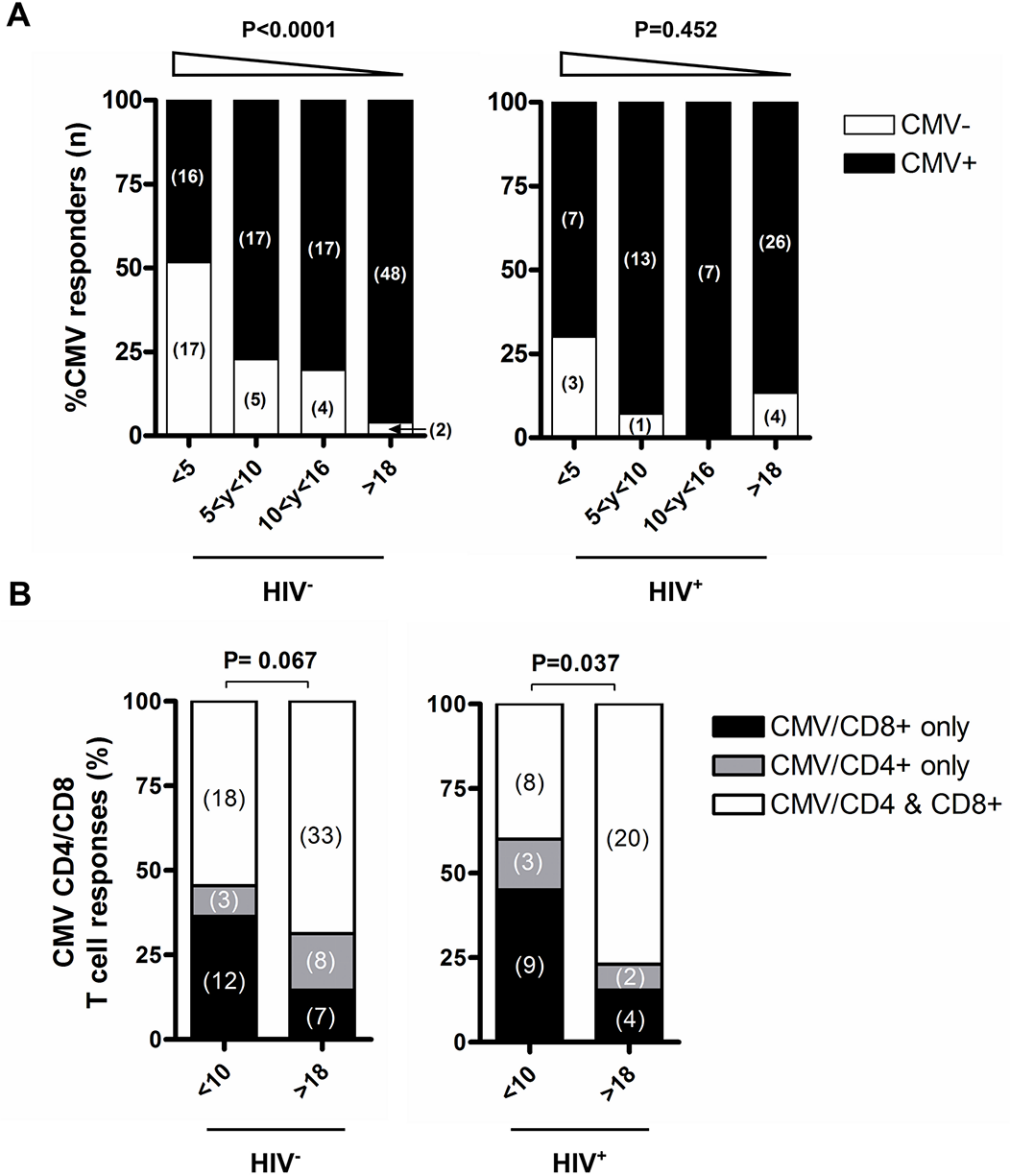
CMVpp65-specific T cell responses in relation to age and HIV infection status. (A) Frequency of subjects with or without CMVpp65-specific T cell responses (CD4^+^ or CD8^+^) is shown stratified by age group and HIV status. B) The frequency of subjects with CMVpp65-specific T cell responses distributed within the CD4^+^ and/or CD8^+^ T cell compartment is shown after stratification by HIV status. The number of subjects in each group is indicated within brackets. CMVpp65-specific T cell responses were considered positive if the frequency of IFNγ^+^ T cells was 2-fold above the negative control and at least 0.05% of the parent population. Statistical analysis was performed using the chi-square test for trend (A) and chi-square test (B).

As expected, the prevalence of CMVpp65-specific T cell responses increased with age (Fig 2A). In the HIV^-^ group, CMVpp65-specific T cell responses were detectable in 48.5% (16 of 33) of children <5 years of age, in 77.3% (17 of 22, Fig 2B) of children between 5 and 10 years, in 81% (17 of 21) of adolescents (10–16 years) and 96% (48 of 50) of adults (Fig 2A, P<0.0001). In the HIV^+^/AIDS group, CMVpp65-specific T cell responses were detectable in 70% (7 of 10) of children <5 years of age, in 92.8% (13 of 14) of children (5–10 years), in 87.5% (7 of 8) of adolescents (10–16 years) and 89.7% (26 of 29) of adults. Interestingly, in children <10 years of age, HIV infection was associated with an increased prevalence of CMVpp65-specific T cell responses (p = 0.067). These data suggest that within the studied Tanzanian population, CMV infections are mostly acquired within the first 10 years of life with HIV infection being a potential risk factor for early acquisition of CMV infection. When compared to West-African populations, CMV infection seems to be acquired at an older age where by the age of 12 months close to 100% of children showed serological evidence of CMV infection or had released CMV DNA in urine [18,19].

CD4 T cell responses targeting purified protein derivatives (PPD) followed a similar trend (data not shown), indicating that detection of Mycobacteria-specific T cells in adolescents and adults are not a consequence of BCG vaccination during early infancy (BCG vaccination coverage for patients <18 years was 97.7% (43 of 44, unknown status n = 1). These findings are consistent with the disappearance of circulating Mycobacteria-specific CD4 T cell responses upon BCG vaccination within one year following vaccination [20].

Next, we compared the contribution of CD4^+^ or CD8^+^ T cells to the CMV-specific T cell responses in children (<10 years) and adults (>18 years). A high number of children (36.4% for HIV^-^ and 45% for HIV^+^) mounted CMV responses exclusively within the CD8^+^ T cell subset irrespective of their HIV status. In HIV^+^ individuals, the difference between children and adults reached statistical significance with more individuals having both CD4^+^ and CD8^+^ CMV-specific T cell responses in the adult HIV^+^ group (Fig 2B, P = 0.037).

**Fig 3:**
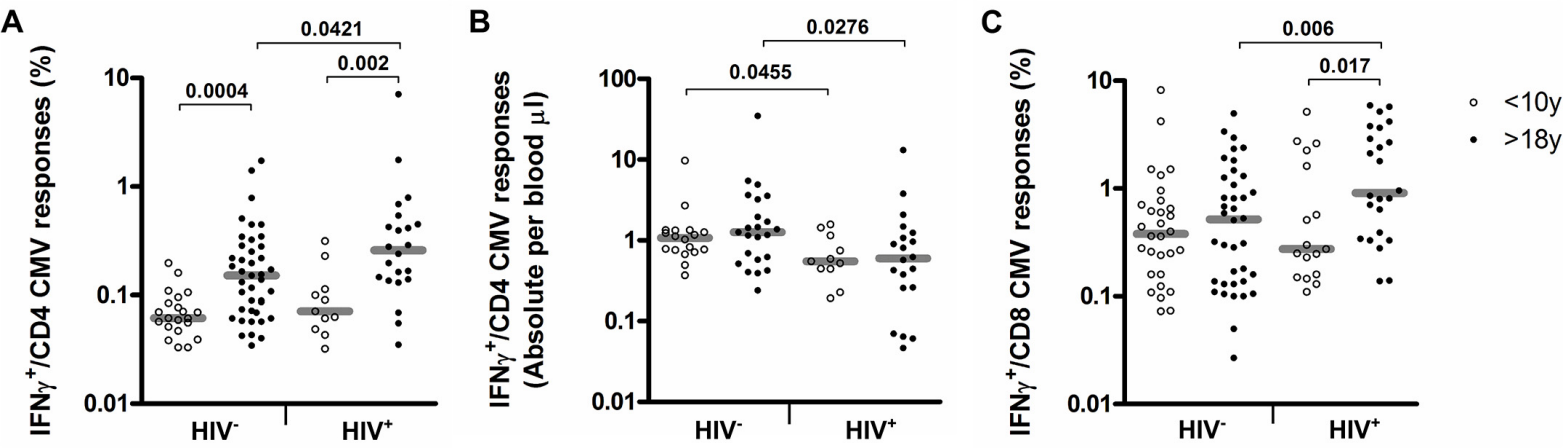
Frequencies and absolute counts of IFNγ^+^-CMVpp65–specific CD4^+^ or CD8^+^ T cell in children below 10 years of age and adults. Scatter-plots depicting A) the frequencies or absolute counts of CMV specific IFNγ^+^-CD4^+^ T cells s and C) frequencies of CMV specific IFNγ^+^-CD8^+^ T cells in the two-age groups and according to HIV status.

**Fig 4:**
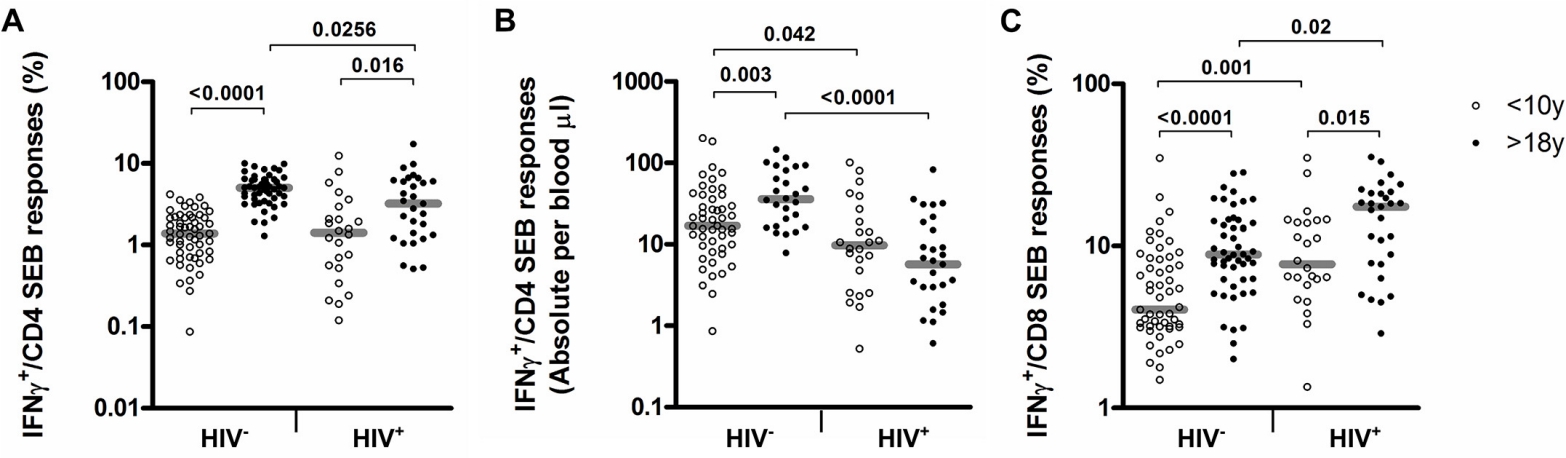
Frequencies and absolute counts of IFNγ^+^-SEB–specific CD4^+^ or CD8^+^ T cell in children below 10 years of age and adults. Scatter-plots depicting A) the frequencies or B) absolute counts of SEB specific IFNγ^+^-CD4^+^ T cells s and C) frequencies of SEB specific IFNγ^+^-CD8^+^ T cells in the two-age groups and according to HIV status.

Adults showed increased frequencies of CMVpp65-specific CD4^+^ T cells compared to children <10 years regardless of their HIV infection status (Fig 3A, HIV^-^: median 0.15% vs. 0.06%, P = 0.0004; HIV^+^: median 0.26 vs. 0.07%, P = 0.002). A higher frequency of CMVpp65-specific CD8^+^ T cell responses in adults was also observed, particularly within the context of HIV infection (Fig 3C, median 0.15% vs. 0.37%, P = 0.017) [4,21]. Absolute counts of CMVpp65-specific CD4^+^ T cells no longer increased with age and were significantly affected by HIV co-infection in children and adults (Fig 3B, P = 0.045 and P = 0.0276 respectively). In line with previous reports showing age related differences in the proportion of IFNγ^+^ T cells in response to SEB stimulation [22–24], we also observed significantly reduced frequencies and absolute counts of IFNγ^+^-CD4^+^ T cell responses after SEB stimulation in children (Fig 4). This could reflect the higher proportion of naïve T cells in children, that cannot express IFNγ [25] and might also be linked to IFNγ promoter hyper methylation of naïve T cells in infants [26].

**Fig 5:**
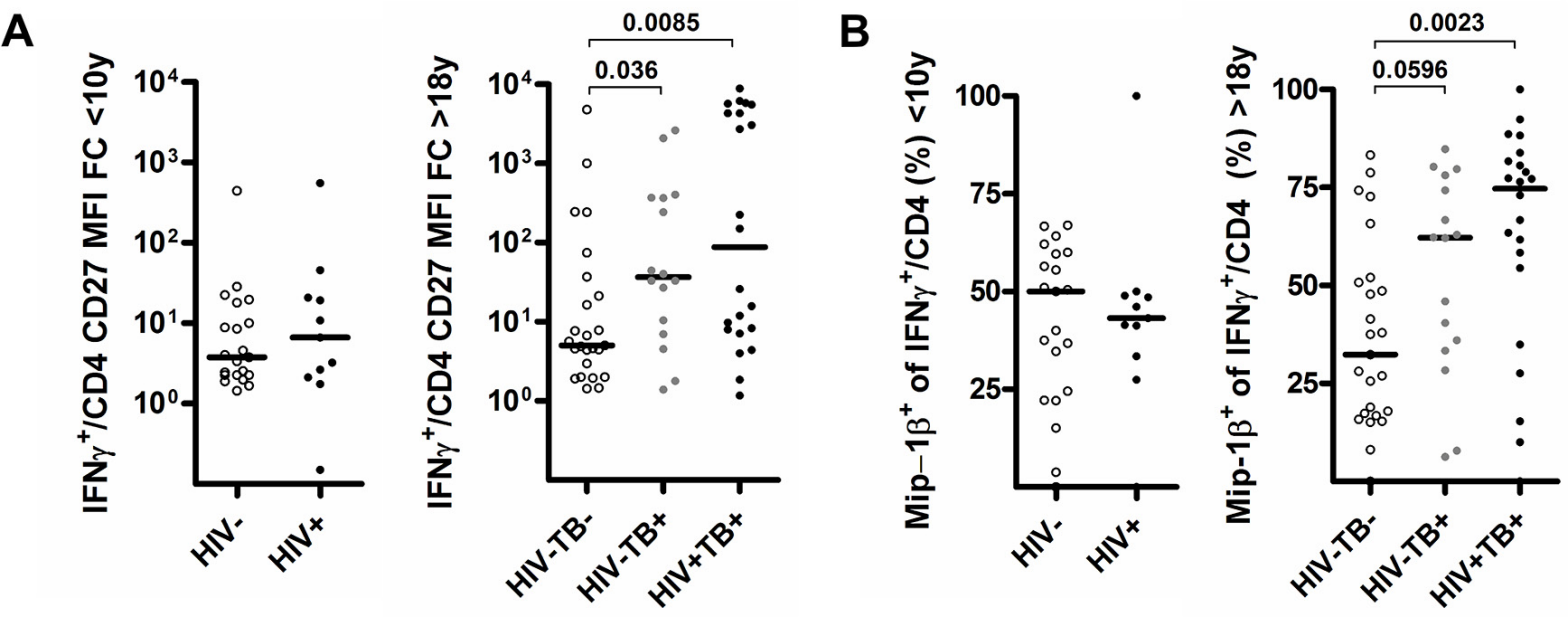
CD27 and Mip-1β expression of CMV specific IFNγ producing CD4 T cell responses are influenced by HIV as well as tuberculosis co-infection in adults. Scatter-plots illustrating (A) the CD27 MFI fold-change to all CD4^+^ T cells and B) the proportion of cells producing Mip-1β of CMV specific IFNγ^+^-CD4^+^ T cell in patients under 10 or over 18 years old and according to HIV and TB infection status.

**Fig 6:**
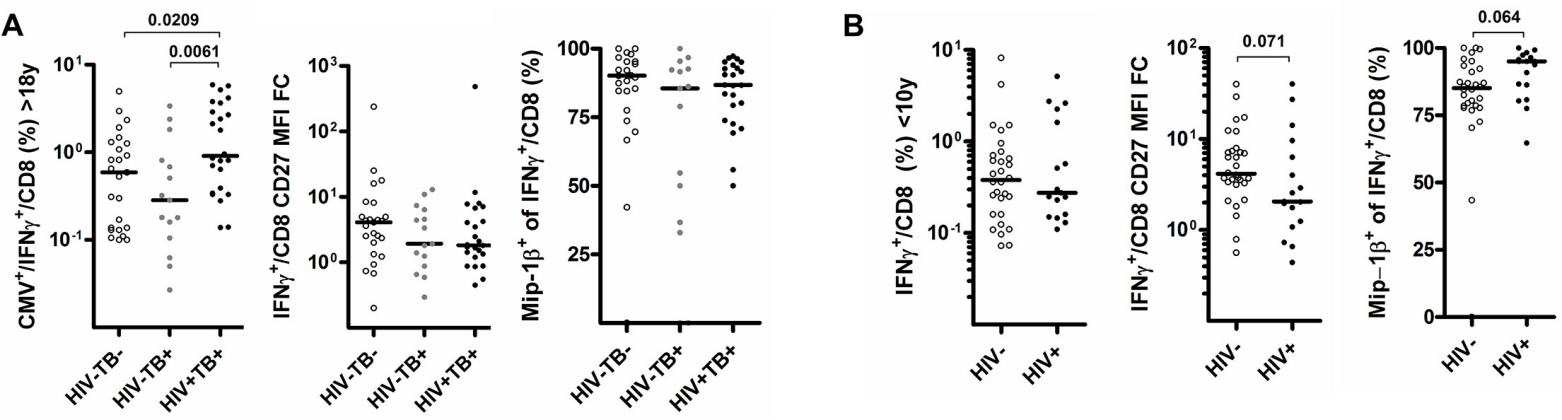
CD27 and Mip-1β expression of CMV specific IFNγ producing CD8 T cell responses are not significantly influenced by HIV as well as tuberculosis co-infection in adults. Scatter-plots illustrating (A) the CD27 MFI fold-change to all CD8^+^ T cells and B) the proportion of cells producing Mip-1β of CMV specific IFNγ^+^-CD8^+^ T cell in patients under 10 or over 18 years old and according to HIV and TB infection status.

Importantly, the proportion of responders with CMVpp65-specific CD4^+^ T cells was similar between HIV^+^ (12 of 19, 63.1%) and HIV^-^ (11 of 20, 55%) children (P = 0.53). This demonstrates that CMV-specific CD4^+^ T cells persisted in children <10 years of age during concurrent HIV infection as described previously for adults [10,27], even though absolute numbers of CMVpp65-specific CD4^+^ T cells were reduced in HIV^+^ adults and children (Fig 3B). Relative resistance of CMV-specific CD4^+^ T cells to HIV-induced depletion has been linked to their high capacity for Mip-1β secretion [7] together with their mature phenotype, characterized by a high proportion of cells expressing the senescence marker CD57 [28]. Expression of CD57 on CMV-specific CD4^+^ T cells correlates with an increased capacity to produce Mip-1β [10]. We thus wanted to determine whether the capacity for Mip-1β production is associated with aging in our cohort. There was no linear relationship between age and capacity for Mip-1βproduction in IFNγ^+^ CMV-specific CD4^+^ T cells in HIV^-^ subjects (data not shown). Both HIV^-^ adults and children had IFNγ^+^ CMV-specific CD4^+^ T cells characterized by a CD27^low^ phenotype (P = 0.332) and a similar capacity to co-express Mip-1β (median 32.4% v 50%, P = 0.372, Fig 5 and Fig 1C and 1D for gating strategy details). A previous study reported a predominance of undifferentiated CD27^+^ CMV-specific CD4 T cells in infants [29] and an unusual immature CD27 phenotype predominance in adults contrasting with several other reports [10],[30],[31]. Differences in study population or methodology could account for this discrepancy. Interestingly, down regulation of CD27 and increased frequencies of Mip-1β co-producing CD4^+^ T cells occurred in adults with active TB (P = 0.036 and 0.0596) and particularly in those co-infected with HIV (P = 0.008 and 0.002). This result demonstrates to our knowledge for the first time that active TB infection alone is associated with phenotypic and functional changes in CMV-specific CD4^+^ T cell response in adults. A significant impact of HIV and active tuberculosis on the CD27 phenotype of CMV-specific CD8^+^ T cells was not observed (Fig 6A and 6B). Based on these results, we hypothesis that that the CMV virus might reactivate during active TB, particularly so in HIV^+^ subjects – driving further antigen-driven CMV-specific cellular maturation and increased Mip-1β production [9,10]. This is supported by the known hematopoietic niche for latent CMV viruses which reactivate upon differentiation of myeloid precursor into tissue macrophages [32]. Cellular recruitment including monocyte-derived macrophages is essential for the granuloma formation that typifies tuberculosis pathogenesis [33]. Similar changes were not detected in HIV^+^ children. However, the number of children included in the analyses and the fact that all enrolled children were TB suspects limits the validity of this finding.

## Conclusion

We show that CMVpp65-specific T cell responses are detectable in the peripheral blood of most Tanzanian children indicating that acquisition of CMV infection typically occurs during infancy and early childhood with HIV being a risk factor. In comparison to adults, there was an increased proportion of CD8^+^ T cells participating in the response and this was particularly apparent in HIV^+^ children. In children, CMVpp65-specific CD4^+^ T cells had a mature CD27^low^ effector memory phenotype and a high capacity for Mip-1β production similar to those detected in adults, and there was no apparent effect of HIV co-infection. Despite some limitations of the current study, such as the hospital based inclusion of children, our results suggest that the high capacity for Mip-1βproduction of CMV-specific CD4^+^ T cells is independent of age. Importantly, we observed for the first time that active TB impacts on the CMV-specific CD4^+^ T cell phenotype. This may reflect recent CMV reactivation and would indicate a previously unknown interplay between CMV virus infection and active tuberculosis pathogenesis.

## Materials and Methods

### Ethics Statement

The study was conducted at two Tanzanian research sites—the NIMR-Mbeya Medical Research Center, Mbeya, and the Ifakara Health Institute, Bagamoyo. The Institutional Review Board of the Ifakara Health Institute, the Mbeya Medical Research and Ethics Committee, and the Medical Research Coordinating Committee of Tanzania approved the study protocols. We obtained written informed consents from adults or for children from a literate parent or legal guardian who participated in the TB child study [17] or the Worm HIV Interaction study (adults only) [34].

### Study subjects and blood sampling

Children older than 7 years provided assent for participation. Children and adolescents (≤16 years old) were enrolled in the context of clinical consultation for illnesses that resemble active tuberculosis. HIV^-^/TB^-^ healthy controls and MTB culture-confirmed TB cases were enrolled into the adult group (≥18 years old). Blood was collected into Vacuette CPDA tubes and PBMC isolation performed using Ficoll gradient centrifugation (Leucosep, Greiner Bio-One GmbH) before cryopreservation in fetal calf serum containing 10% DMSO within 6h of phlebotomy.

### Reagents

Complete Medium (CM) (RPMI 1640 W/Glutamax and Penicillin-Streptomycin (Gibco, Invitrogen), 100μg/ml, 10% heat-inactivated FCS (FBS Gold A11-151, PAA Germany). Anti-CD4-PerCP Cy5.5 (Oct4) and HLA-DR Pe-Cy7 (LN3) were purchased from eBioscience. Anti-CD8 Horizon V500 (RPA-T8); anti-CD27-APC H7 (M-T271); anti-CD3-Pac Blue (UCHT-1); anti-IFNγ-FITC (B27); anti-CD154-APC (TRAP1); anti-Mip-1β-PE (D21-1351); CST and compensation beads were obtained from BD Pharmingen.

### Intracellular cytokine staining

PBMC were thawed and washed twice in CM containing benzonase (Novagen, 25KU, 1/5000 V/V). Viability was assessed using trypan blue exclusion after a resting period of 2–6 hours at 37°C in complete medium. Cells were stimulated for 12h to 16h in complete medium containing Brefeldin A (Sigma-Aldrich, 5ug/ml) and CD28/CD49d antibodies (Pharmingen, 1ug/ml) with CMVpp65 peptide set (JPT Peptide Technologies, 2ug/ml), Staphylococcal Enterotoxin B (Sigma-Aldrich, 0.8ug/ml) or nothing as a negative control. Intracellular cytokine staining was performed using a standard staining protocol [10]. Acquisition of cells was performed on BD FACS Canto2 (NIMR-MMRC, Mbeya) or BD Fortessa (IHI-BRTC, Bagamoyo). Instruments were calibrated before each run using BD Cytometer Setup and Tracking Beads according to manufacturer’s recommendations. Data were analysed using FlowJo 9.X (Tree Star). T cell responses were considered positive, if the frequency of IFNγ^+^ cells was above 0.05% of the parent population and >2-fold the background frequency in the unstimulated control. GraphPad Prism (San Diego, CA, USA), version 4.03, was used for statistical analysis. Anonymized patient characteristics and the respective flow cytometry data statistics are provided as S1 Table.

## Supporting Information

S1 Table Participant level data; anonymized patient characteristics and respective flow cytometry data statistics. Column legends: A) Tuberculosis classification (see [17]); B) Age (years); C) HIV status (1: positive, 2: negative); D) CD4 cell counts per μl of blood; E) CD4 T cell percentages observed after PBMC stimulation; F) Signif: CD4 T cell response to CMVpp65 stimulation passed threshold for significance (see methods), NS: Not significant; G to I) Percentages of CD4 T cell producing one or both of the indicated cytokine(s) observed after PBMC stimulation with CMVpp65 (γ: IFN-γ, MIP: Mip-1β); J) Proportion of CD4 T cell producing Mip-1β that also produced IFN-γ; K) CD27 MFI Fold Change of IFNγ producing cells compared to all CD4 T cells (see Fig 1 for gating strategy); L to Q) as for F to K) but for CD8 T cell responses to CMVpp65 PBMC stimulation.
(XLSX)
